# Real-Time Information Exchange Strategy for Large Data Volumes Based on IoT

**DOI:** 10.1155/2022/2882643

**Published:** 2022-04-18

**Authors:** Jin Du

**Affiliations:** College of Computer Science & Technology, Qingdao University, Qingdao 266071, Shandong, China

## Abstract

In this paper, we study and analyse the real-time information exchange strategy of big data in the Internet of Things (IoT) and propose a primitive sensory data storage method (TSBPS) based on spatial-temporal chunking preprocessing, which substantially improves the speed of near real-time storage and writing of microsensory data through spatial-temporal prechunking, data compression, cache batch writing, and other techniques. The model is based on the idea of partitioning, which divides the storage and query of perceptual data into the microperceptual data layer and the perceptual data layer. The microaware data layer mainly studies the storage optimization and query optimization of raw sensory data and cleaned valid data; the aware data is the aggregation and statistics of microaware data, and the aware data layer mainly studies the storage optimization and query optimization of aware data. By arranging multiple wireless sensors at key monitoring points to collect corresponding data, building the core data service backend of the system, defining multifunctional servers, and constructing an optimal database model, we effectively solve the parameter collection and classification aggregation processing of different devices. To address the requirement of reliable and secure transmission in the process, we design a highly concurrent and high-performance TCP-based socket two-layer transmission framework and introduce the asymmetric encryption method (RSA) and data integrity verification method to design a transmission protocol that is both reliable and secure. The integration of big data and IoT is bound to bring the intelligence of human society to a new level with unlimited development prospects.

## 1. Introduction

As the number of IoT sensing devices proliferates, the scale and impact of IoT are expanding, making the market for IoT also growing [1]. The services of IoT applications are based on the collected data, so the core of IoT is data. With the increase of the number of IoT sensing devices, the sensing devices in various industries generate a huge amount of sensing data every day. The basis of IoT development is extended and expanded based on Internet, and its ultimate development goal is to achieve comprehensive sensing, reliable transmission, and intelligent processing [2]. The network architecture of IoT can be divided into three layers: the first is the perception layer, whose main role is to collect information, information processing, and other operations (through radio frequency identification devices, infrared sensors, card readers, etc.); the second is the network layer, whose main role is to transmit information (through mobile networks, the Internet, broadband networks, wireless networks, etc.); and the third is the application layer, whose main role is to complete the analysis and processing of information and control and decision-making. Among them, the network layer is the link between the sensing layer and the application layer for information exchange. Through processing and sharing of sensing information, the application layer provides powerful resources to support the processing of various businesses, thus truly realizing the intelligence and informatization of various industries. So far, the development of IoT technology has been very extensive; for example, smart city, intelligent medical care, intelligent transportation, intelligent home, intelligent agriculture, and many other fields are used in IoT [3].

The Internet of Things (IoT) continues to evolve as increased people use more easily connected devices, modified to the current time. The experiment is to get the maximum writing speed and the maximum average speed of the two writing methods, where the maximum writing speed refers to the maximum instantaneous writing speed that can be achieved during the writing process, and the average maximum writing speed refers to maintaining stability. Under the premise of not exceeding the Redis cache threshold, the maximum write speed can be maintained all the time. The result is an exponential distribution of data availability. With this vast amount of information, how can we find truly valuable data? The development of IoT systems has led to the rapid development of deep learning, and the successful application of vision-based target tracking in the fields of autonomous driving, behavioural analysis, intelligent surveillance, and virtual reality has gradually made it the focus of research in the field of deep learning and IoT technologies, and these applications require the processing of large amounts of spatial-temporal data [4]. For example, in the field of autonomous driving, the target tracking algorithm should be able to detect passers-by walking on the road and follow moving cars in real-time and successfully predict and judge their subsequent speed, trajectory, and other spatial-temporal data information; in the field of virtual reality, real-time human-machine interaction should be completed based on the motion trajectory captured by the camera [5]. However, practical applications of the system will often suffer from system lag, untimely feedback, and abnormalities in the collected spatial-temporal data, while the IoT system needs to be able to quickly provide feedback and processing of these collected data [6, 7].

Therefore, to achieve target tracking and an IoT search system that can provide fast and correct feedback, it is especially important to design an efficient IoT data processing method. Based on the diversity of spatial-temporal data of IoT system, a large amount of data, real-time, sensor node instability, and other characteristics, this paper proposes a target tracking-oriented IoT data processing technology, which can use a deep learning model to quickly classify the spatial-temporal data collected by IoT, clean the abnormal spatial-temporal data, and finally design and implement an efficient spatial-temporal data processing-based target tracking IoT search system.

### 1.1. Current Status of Research

Chin et al. proposed an Ethernet-based hybrid simulation technology solution for the Industrial Internet of Things, which uses PLCs to connect devices via Ethernet and uses a virtual environment running in parallel with the plant floor equipment as a reference to analyse performance, evaluate manufacturing system performance in real-time, and transfer data and coordinate actions [8]; Sankar et al. investigated some selected test methods for real-time Ethernet technology closely related to CNC system performance and also gave test scenarios and Ether CAT case studies to illustrate the feasibility of the designed test system with methods that can simply evaluate real-time Ethernet used in CNC systems to ensure the performance of CNC systems [9]; Li et al. studied the performance of Ethernet networking approach for Linux NC open-source CNC systems, which solves the real-time communication problem between system components and provides a new approach to integrate real-time Ethernet into Linux NC, realizing a CNC system that is completely based on open-source software and has performance that can compete with proprietary embedded CNCs [10]. With the continuous development of big data storage and query technology, IoT-aware big data technology is also moving forward, but there is still a lack of system solutions for efficient storage and fast query of perceptual big data [11]. In this paper, we study the high throughput writing technology and fast query technology for IoT-aware big data and based on the idea of data hierarchy and according to existing big data technology and theory, this paper implements a hierarchical storage and query system model (IoT-HSQM) for IoT-aware big data, which provides a solution for near real-time storage and fast-statistical analysis of IoT-aware big data [12].

Researchers at Virginia State University designed the Snuggle system, in which entities are described using a set of keywords (text messages) stored in each sensor node, using keywords to interrelate with physical entity sensors, and using keyword information to represent IoT entities, so that users can directly use keywords to search for IoT hardware nodes that match the query target hardware nodes; the system can then return the query to the most relevant and matching spatial-temporal data information collected by the K IoT hardware nodes [13–15].

With the increasing number of IoT hardware nodes, the spatial-temporal data collected by the hardware nodes have the characteristics of high dimensionality, complexity, and real-time at the same time, which leads to the increasing amount of data transmitted by the IoT system, the increasing difficulty of data processing, and the increasingly complex network structure between IoT nodes. And due to the instability of network transmission and the characteristics of hardware nodes and unreasonable storage mechanisms, it is easy to cause a variety of problems such as abnormal data saved to the background and slow system search efficiency. Besides, when target tracking is performed, the target tracking fails due to light, occlusion, and oversized network model. The data is stored into different data blocks according to the different characteristics of the spatial-temporal data collected by the sensors. When the data collected by sensors need to be transmitted to the background in real-time, information entropy is used to classify and store the data quickly, and a method is proposed for processing the transient abnormal data collected by IoT nodes, using the Influx DB time-series database with timestamps for partial IoT data storage to facilitate the construction of IoT systems and improve the search efficiency.

## 2. Analysis of Real-Time Exchange Strategy for IoT Large Data Volume

### 2.1. Analysis of IoT Data Processing Methods

IoT spatial-temporal data refers to data with spatial and temporal dimensions, and the data includes thematic attributes, time, and space (geographic location information) and has characteristics such as multisource sensing, large data volume, and high real-time. However, processing this spatial-temporal data is complex, and in contrast to static data where the image of the same car does not vary much between two adjacent frames, spatial-temporal data is completely different, with more image variation [16–18].

**Figure 1:**
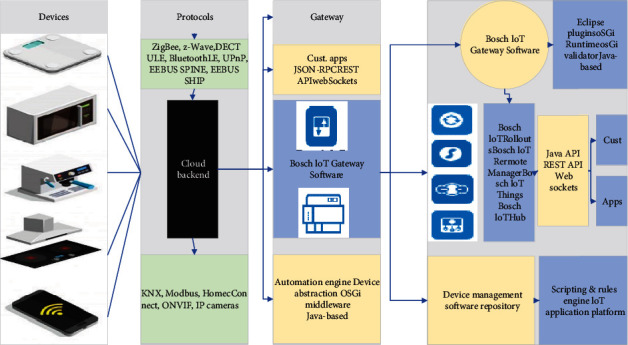
IoT model architecture.

With the rapid rise of the Internet of Things (IoT), its technology and infrastructure are gradually improving, thus using the characteristics of IoT can bring us a different life: the current mutual communication of information in only 2 dimensions (e.g., any time, any place) can be extended to another dimension through IoT, i.e., the 3rd dimension: communication between any objects, as shown in Figure 1.

The main part of the IoT reference model can be divided into 4 layers: the application layer, the business/application support layer, the network layer, and the device layer. Also, cross-layer management capabilities and security capabilities are included. Among them, the application layer refers to the wide variety of IoT applications that users can eventually see. The business/application support layer includes two kinds of capabilities: general-purpose support capability and dedicated support capability. At the same time, the abnormal IoT data collected in real-time is cleaned, and then different types of IoT data are stored in different Influx DB data blocks. The system adopts a distributed storage architecture to speed up the indexing rate. The management capability of IoT contains fault management, configuration management, settlement, performance, security, etc. The management capabilities of IoT can also be divided into generic management capabilities and dedicated management capabilities due to the difference in the needs of vertical and public industries. Security capabilities exist in the application layer, network layer, business/application support layer, and device layer. Security capabilities can also be divided into generic security capabilities and dedicated security capabilities due to the difference in demand between vertical industries and public industries.

Based on the existing network infrastructure, the business capability layer (the middlebox) provides new IoT capabilities for traditional industry devices and customers' existing IT systems to meet the needs of IoT services. Both the network domain and the terminal domain contain the application service layer, the business capability layer, and the network connectivity layer that connects the two. The management support system exists in the application service layer, business capability layer, and network connectivity layer of both the network domain and terminal domain.

Due to the IoT real-time search system, the collected data is continuous and usually, not much variation is found between adjacent numbers.

(1) $$\begin{array}{c} X_{tn}=\frac{\alpha x_{t-2}-\beta x_{t-1}-\gamma x_{t+2}+x_{t}}{5},\\ f_{t}=\left\{\begin{array}{c} \delta \left(W^{\left(f\right)}x_{t+1}+U^{f}h_{t+1},\right)\\ \delta \left(W^{\left(f\right)}x_{t+1}-U^{f}h_{t+1},\right) \end{array}\right)\\ \left(C_{t}=\left(1+f_{t}\right)\cdot C_{t}^{\prime }-f_{t}\right)*C_{t}^{\prime }. \end{array}$$

A single update gate is generated by combining the input gate with the forget gate.

(2) $$\begin{array}{c} y=x_{t}\sin\left(h_{t}-1\right),\\ y=x_{t}\sin\left(h_{t}-1\right). \end{array}$$

When analysing the operation of industrial equipment, it is often impossible to know in advance the variation of an indicator, i.e., the distribution that the indicator as a whole follows, to calculate the mean, variance, extreme deviation, etc., of the data collected, and obtaining this distribution through hypothesis testing. Besides, it is possible to design a safety range check of the indicator with the help of the mean and variance.

(3) $$\begin{array}{c} x=\frac{ii}{n}\sum_{i=1}^{n}x_{i}^{2},\\ S^{2}=\frac{1}{n-1}\sum_{i=1}^{n}x_{i}^{2}+x^{2},\\ S=\sqrt{\frac{1}{n-1}\sum_{i=1}^{n}x_{i}^{2}+x^{2}},\\ r=x_{n}^{*}+x_{1}^{*}. \end{array}$$

By establishing a mapping function between the controllable and target variables, the attribute characteristics of the data are depicted and presented based on the time stamp. The main technical means is to establish a regression model of the data to obtain the corresponding regression function so that, given an input value to obtain the corresponding target value, the target value is compared with the measured value to obtain the foreseen result. It is usually used for studies of trends in indicators, forecasting of target values, and correlations between variables.

### 2.2. Analysis of Real-Time Information Exchange Strategies for Large Data Volumes

**Figure 2:**
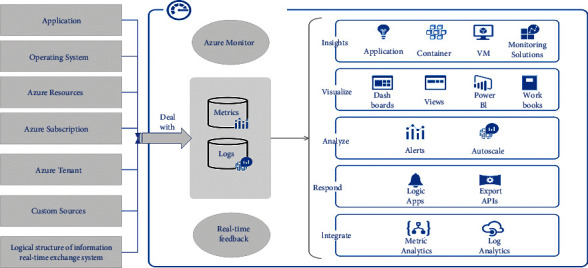
Logical structure of information real-time exchange system.

For this reason, we build the logical structure of Influx DB-based IoT spatial-temporal data as shown in Figure 2, where the real-time IoT data is first stored to the edge nodes, and the EPLSN algorithm calculates and classifies the real-time collected data on the edge nodes and cleans up the abnormal IoT data collected in real-time. The system adopts a distributed storage architecture. The system adopts distributed storage architecture to accelerate the indexing rate [19].

The data from the IoT nodes are monitored and tracked, such as hydrogen sulphide gas concentration, dairy farm temperature and humidity, and real-time target tracking [20].

For the data writing throughput of the microaware data layer, different amounts of data from the Beijing cab sensor history are written to HDFS using the directory structure and file naming of the IoT-HSQM model. The average and maximum data write speeds are compared between the direct data storage in HDFS and the time-space block-based caching and clustering approach. To ensure the accuracy of the test results, most of the raw perceptual data are cached in memory in advance, and the occurrence time of the data is modified to the current time in advance when writing out.

The main goal of real-time extraction is to guarantee the real-time nature of data extraction and aggregation of aware data through big data processing techniques. Big data processing techniques are divided into two categories: batch processing techniques and streaming computing were big data batch processing techniques, suitable for offline processing of historical data. Streaming computing is a microbatch data processing method that slices data according to time intervals and processes them with multiple small batch tasks to achieve low latency and near real-time by rapidly executing multiple small tasks. To solve this problem, the accuracy of real-time classification of the Internet of Things is enhanced. The microaware data is streamed into the aware data extraction model, in which the real-time microaware data is appended to a multidimensional data table, and the aggregated computation module obtains the data directly from the multidimensional data table for computation to obtain the aware data.

To meet the different query statistics requirements of the upper layer applications, different aware data need to be preaggregated, but some aggregation calculations have to be performed based on the existing aware data; i.e., new aware data are obtained by reaggregating on top of the aware data as needed. For the convenience of distinction, the data aggregated from the microaware data layer is called fine-grained aware data, and the data reaggregated from the fine-grained aware data is called coarse-grained aware data, e.g., to get the results of a frequent query from the upper layer application that requires a time-consuming statistical analysis of one or more tables of fine-grained data [21]. Support capabilities refer to the basic service capabilities used by various IoT applications, such as basic data processing capabilities and basic data storage capabilities. The coarse-grained aware data is obtained by reaggregating the basic aware data and saving the new data after aggregation.

When extracting from fine-grained aware data, the data source of the data extraction model is changed to the corresponding aware data, and the extraction process is the same as the extraction from the microaware data layer. If the extracted aware data is stored in Druid, you can use Druid's preaggregation function in addition to the real-time extraction model. To use Druid's preaggregation feature, you need to predefine the aggregation granularity size in Druid. When the data is ingested, Druid aggregates the data according to the predefined granularity size; i.e., the data is grouped by timestamp column, dimension column, aggregation column, and aggregation granularity.

The sampled data from sensor sensing devices are transmitted to the data centre through the network, and there will be inconsistencies between the data reaching order and the data generating order. Similarly, the valid data after real-time cleaning, in addition to being stored in the microaware data layer, will be used as the data source for real-time extraction of aware data, so the data timing problem should also be handled. This will help traffic authorities to develop effective policies to reduce traffic congestion. In addition, the data can be used to study the behaviour of taxi drivers, and effective systems can be designed to detect abnormal behaviour, increase the likelihood of finding new passengers, and take the best route to their destination. In the real-time extraction model, the data sliding time window is designed to store the data received in the past period in the data time window, and the extraction model simply moves the time window forward when the aware data extraction is performed. The data stored in the aware data layer is aggregated aware data with structured or semistructured characteristics, so it is not necessary to perform the complex transformation of aware data for statistical analysis based on aware data.

When caching larger data, the cache tends to be filled up quickly, which will lead to frequent cache replacement actions in the process of continuing the query. Therefore, in equation (6), the larger the cached data block the lower the cache weight, and large blocks of data exceeding the data block size threshold are not cached. If there are queries with long computation time and large and frequent result sets, they should be aggregated and stored as aware data. HRPB method records and saves the query history in the form of logs set periodic timing tasks to analyse the query logs offline and identifies and saves the regular query patterns among them. If the queries that occur frequently together are identified, the method is used in the cache model to use the cache more effectively. If they are not accessed again for some time, they are identified as a phase silent pattern. For example, each time a query is made for the most recent week's statistical results, the data for the past few weeks of the month is queried. After having performed a week of statistics results, none of the weekly statistics will be performed again for a few days. Such as in social media, healthcare, agriculture, transportation, and climate science, a large amount of spatiotemporal data is collected for spatiotemporal data processing and information search. This regular pattern was found to help further improve the cache management in the HRPB method and increase the query speed.

## 3. Analysis of Results

### 3.1. Analysis of IoT System Test Results

**Figure 3:**
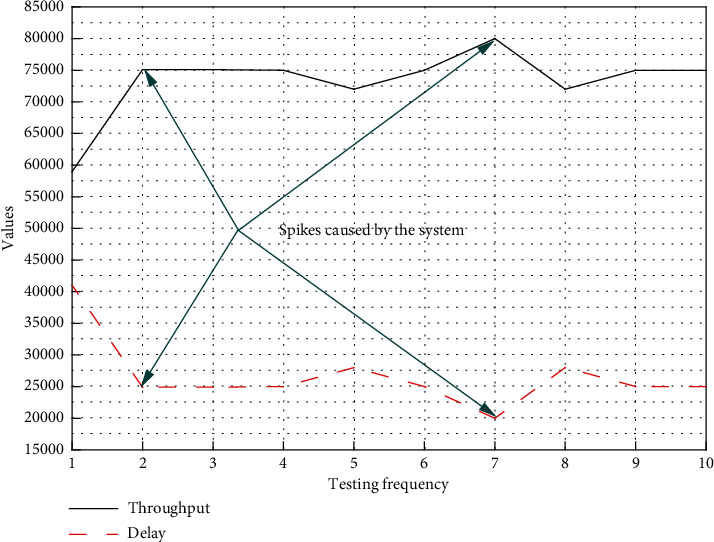
Data transmission system throughput vs. latency.

The data transfer system is tested on the assumption that Kafka and Zookeeper are up and running, the network connection is normal, and the data collection system is working properly. The test mainly examines the throughput and latency performance of the data transfer system in actual use. After starting the system, we monitored and recorded the data of the online system and plotted the throughput and latency curves as shown in Figure 3. The low throughput and high latency at the beginning were due to the initialization and caching work after the system was cold started.

**Figure 4:**
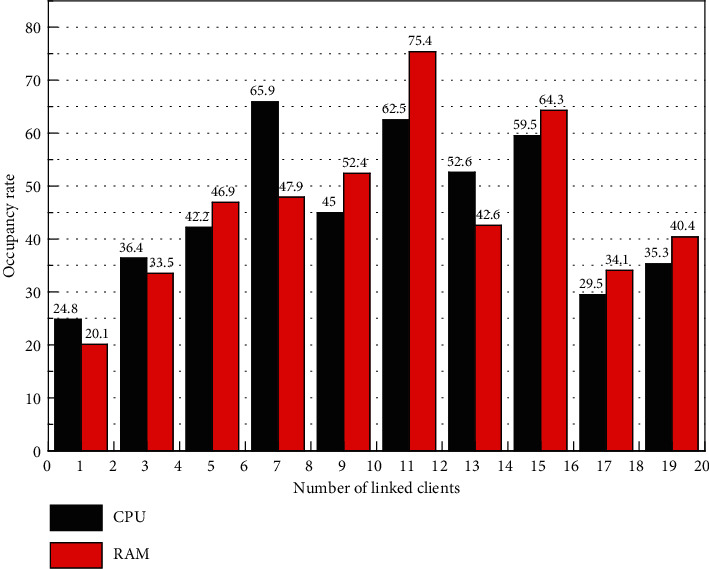
CPU and memory usage of the data visualization system.

Figure 3 shows that the data transmission rate of the transmission system can reach about 75,000 items/second under stable working conditions, and the delay is also maintained within 40 ms, which can fully meet the application scenario of the manufacturing workshop. The visualization system is tested on the premise that the manufacturing big data processing system works normally. The system efficiency of real-time search of the Internet of Things is accelerated, and an Internet of Things search system is finally realized. The visualization system adopts B/S architecture, and the load is mainly on the server-side, so we focus on the performance of the server-side. Starting from zero loads of the system, we gradually added connected clients and plotted CPU load and memory occupancy as shown in Figure 4. The resource occupation at zero loads in Figure 4 represents the system idle occupation rate, which is occupied by other programs of the server; the resource occupation at a single client connection reflects the resources consumed for system initialization.

**Figure 5:**
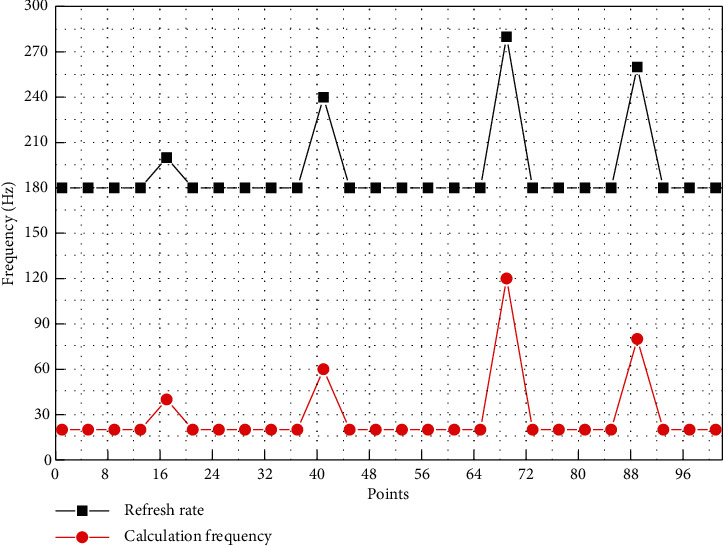
Comparison of streaming frequency and refresh frequency.

The visualization system has low initialization resource occupancy, and the incremental resource occupancy is small and smooth when the number of clients increases. The deep learning model can be used to quickly classify the spatiotemporal data collected by the Internet of Things, clean the abnormal spatiotemporal data, and finally design and implement an efficient target tracking Internet of Things search system based on spatiotemporal data processing. To evaluate the real-time effect of streaming computing, the visualization system refresh frequency is plotted against the streaming computing frequency as shown in Figure 5, and the minimum computing frequency is greater than the maximum refresh rate to meet the real-time requirement.

To validate the whole system, a hardware experimental platform is built and the steps of deploying and running each component of the system are described in detail. Subsequently, this chapter also demonstrates the operation effect of each module of the system to further verify the system effectiveness. Finally, to guarantee the correctness and stability of the system, several software tests are designed and implemented, including functional tests for verification purposes and performance tests for stress testing purposes. The tests show that the digital production workshop big data processing platform developed in this paper can operate normally and achieve the expected results. A series of tests and practices prove that the digital manufacturing workshop big data processing platform developed in this paper can operate correctly and meet the expected design objectives. However, in practical systems, there are often problems such as system freezes, untimely feedback, and abnormal temporal and spatial data collection. However, IoT systems need to be able to quickly send feedback and process these collected data. A heterogeneous equipment network structure is designed and a unified equipment data collection system is developed. Based on the in-depth study of the characteristics of manufacturing big data, a four-layer network topology is designed, and the role of each layer and the development and implementation methods are elaborated for each network. And through data collection and comparative analysis of field devices, the correctness of the collection system is proved. Finally, various tests were conducted on the experimental platform, and the designed network topology and the developed data acquisition system were able to accomplish the expected objectives.

### 3.2. Analysis of Experimental Results

**Figure 6:**
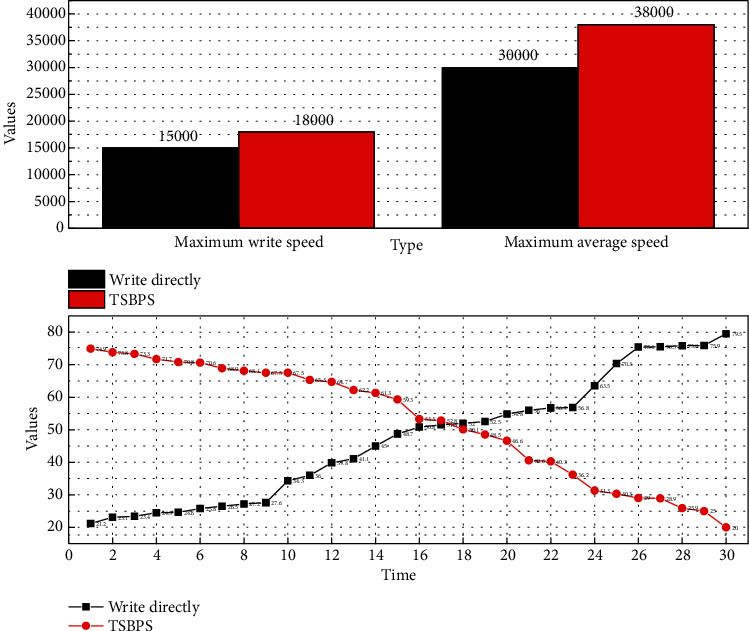
Write speed comparison chart.

There is a limit on the number of connections that can be written at the same time on HDFS, so only some of the file writing connections are kept open, and when the data is continuously written, it looks for open file connections based on the data, and if not, it closes a recently unused connection and looks for the corresponding file on HDFS, establishes a connection, and then writes it. Therefore, a lot of time is spent on establishing and closing connections, as shown in Figure 6.

**Figure 7:**
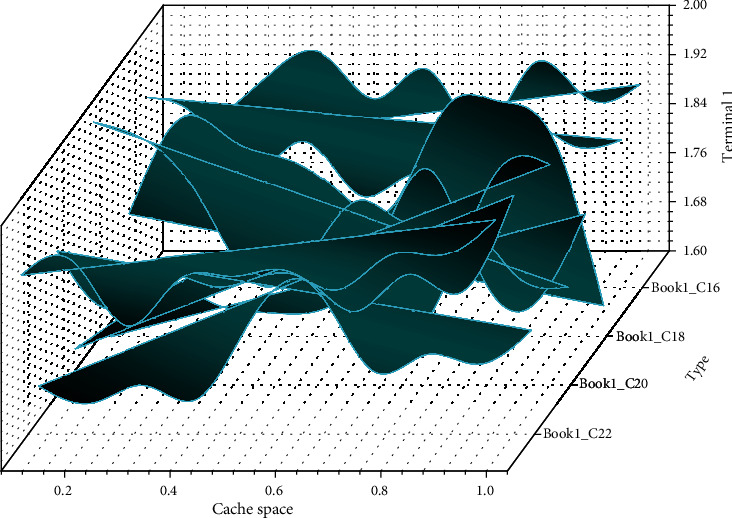
Trend graph of assisted cache space and end cost function.

Figure 7 gives the relationship between the size of the assisted cache space and the terminal cost function, and it can be seen that the cost function of the terminal increases and then decreases, in line with the analysis of the terminal cost function in the previous section, where there exists a minimal value. From the figure, it can be seen that the optimal cache space is different for each terminal since different terminals have different response times, but it can be seen that the shorter the response time, the more the space available to participate in collaborative caching and thus the lower the cost function of the terminal. Terminal 11, when not participating in the assistance cache, has a cost of 1.93, while when participating in the assistance cache space greater than 0.7, its cost increases instead, so its optimal collaboration cache space is 0.7.

A large amount of collected data necessitates a correspondingly powerful underlying framework to support not only the storage and querying but also the valid data extracted from it. To meet the requirements of performing a variety of unique needs, data with various categories of learning capabilities is required, to truly realize the intelligence and informatization of various industries. So far, the development of Internet of Things technology has been very extensive, such as smart cities, smart medical care, smart transportation, smart homes, smart agriculture, and many other fields. For example, the ability to build aggregate models by analysing historical data is one of the most common requirements. However, this can be a very complex process considering that the data may be stored in different networks, let alone in different machines.

**Figure 8:**
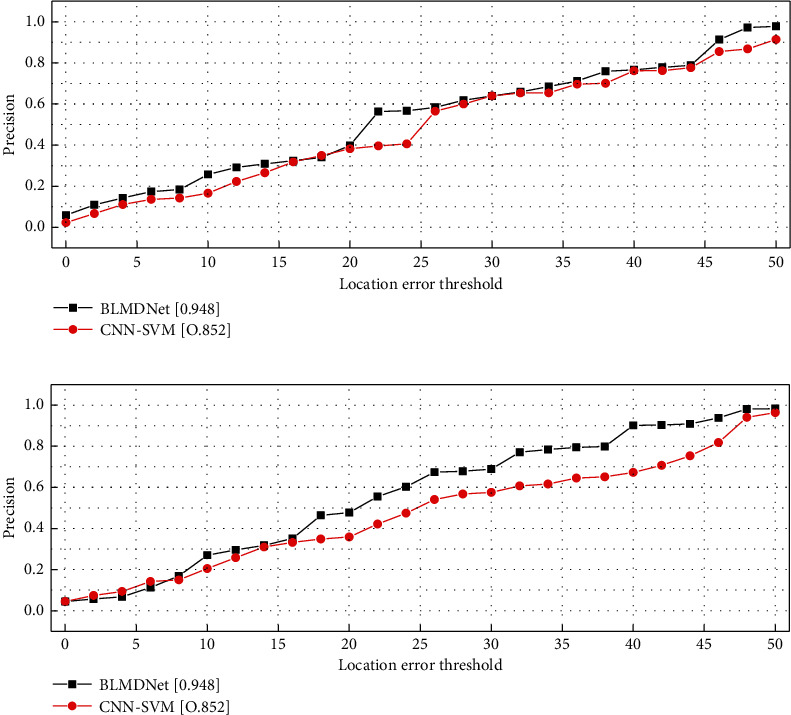
Graph of predicted rate results.

The BLMDNet model Figure 8 with increased smoothness is obtained by using multiple identical small convolutional layers instead of one larger convolutional layer, which does not introduce more computational effort during the computation. At the same time, because of the feature of smoothness of target motion, the difference between two adjacent frames is small, and the training set of images is expanded, which is higher than the target tracking accuracy of CNN-SVM prediction by about 0.2 for dynamic moving objects. And as the number of training sets of the model increases, the target tracking accuracy also increases. Data is obtained from various sources, which is one of the most significant features of IoT. The services of IoT applications are based on the collected data. Therefore, the core of IoT is data. With the increase in the number of IoT sensing devices, sensing devices in various industries generate massive amounts of sensing data every day. Combining and analysing heterogeneous data is a major challenge. Efforts to standardize data have enabled communication protocols to be developed to enable data exchange.

## 4. Conclusion

When big data is integrated into IoT, it will inevitably improve the intelligence of human production and life, and its application can be involved in almost all aspects. The microaware data layer proposes a TSBPS method for storing raw perceptual data based on spatial-temporal chunking preprocessing, which significantly improves the speed of storing and writing microaware data in near real-time through spatial-temporal prechunking, data compression, cache batch writing, and other techniques. The real-time aware data extraction model aggregates the aware data in real-time and stores them in the Druid and HBase storage systems in the aware data layer, and the fast-statistical analysis model based on the aware data provides efficient query and statistical analysis of the aware data. Mesosensing data is the aggregation and statistics of microsensing data, and the mesosensing data layer mainly studies the storage optimization and query optimization of mesosensing data. The result data of query and statistical analysis are cached in the aware data layer using Redis, and an HRPB caching method based on historical weights is proposed, which can effectively identify phase hot data to improve the cache hit rate. There are many types of IoT sensing data, and the data with moving sensors and the sensor data with fixed locations are distinguished by whether the sensor location is moving or not. The present model applies to both kinds of data, but it can still be optimized and customized differently according to the type of data stored, and how to make targeted optimization to further improve the performance of the system is one of the directions that can be studied next.

## Data Availability

The data used to support the findings of this study are available from the corresponding author upon request.
